# Supplementary material for: Identification of photoperiod-induced specific miRNAs in the adrenal glands of Sunite sheep (Ovis aries)
Source: Front Vet Sci. 2022 Jul 22;9:888207. doi: 10.3389/fvets.2022.888207 (PMC9354845; doi:10.3389/fvets.2022.888207)
Supplement: Supplementary file 1 [file Data_Sheet_1.ZIP › Supplementary file/miRDeep2 file.zip!/miRDeep2 file/Novel_Structure/SPLP42A3/Novel_292.pdf]

Provisional ID	:	NC_019459.2_5106
Score total	:	2
Score for star read(s)	:	-1.3
Score for read counts	:	0
Score for mfe	:	1.7
Score for randfold	:	1.6
Score for cons. seed	:	
Total read count	:	805
Mature read count	:	802
Loop read count	:	0
Star read count	:	3

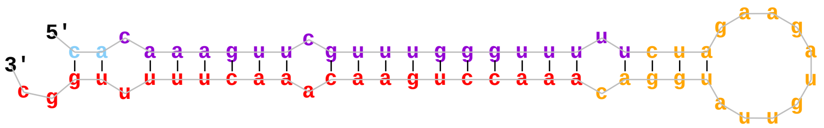

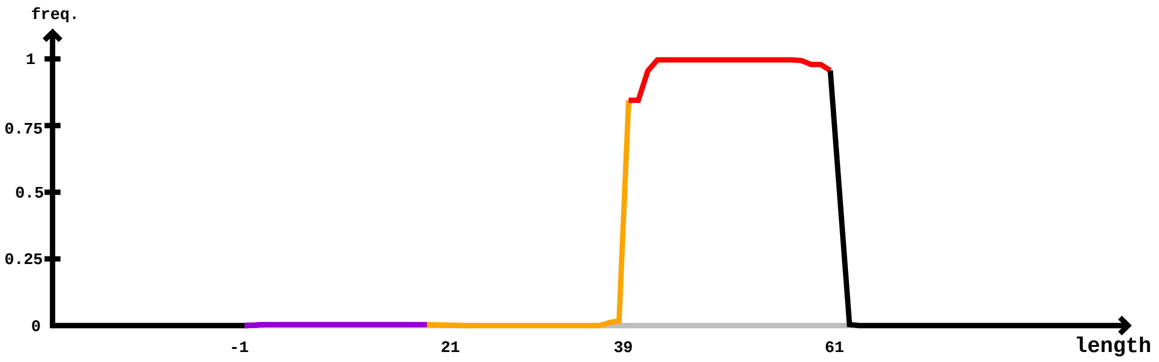

Star   Mature

```
5'- auaugguauuaaauuggccacaaaguucguuuggguuuuucuagaagauguuauggacaaaccugaacaaacuuuuuggccagcccaauaccauuaggugccaucuacua   -3'    obs
    auaugguauuaaauuggccacaaaguucguuuggguuuuucuagaagauguuauggacaaaccugaacaaacuuuuuggccagcccaauaccauuaggugccaucuacua          exp
    ..((((((((...(((((((.((((((.((((((((((.((((..........)))).)))))))))).)))))).)))))))...))))))))(((((...)))))...   reads   mm   sample
    ...................acaaaguuUguuuggguuuuuc.....................................................................   1       1    seq
    ....................caaaguucguuuggguuuuu......................................................................   2       0    seq
    ........................................................aAaaaccugaacaaacuuuu..................................   2       1    seq
    ........................................................aAaaaccugaacaaacuuuuu.................................   7       1    seq
    .........................................................Aaaaccugaacaaacuuuuu.................................   1       1    seq
    .........................................................Aaaaccugaacaaacuuuuugg...............................   4       1    seq
    ..........................................................aaaccugaacaaacuuuuu.................................   1       0    seq
    ..........................................................aaaUcugaacaaacuuuuu.................................   3       1    seq
    ..........................................................aaaUcugaacaaacuuuuugg...............................   14      1    seq
    ..........................................................aaaccugaGcaaacuuuuuggc..............................   1       1    seq
    ..........................................................aaaAcugaacaaacuuuuuggc..............................   2       1    seq
    ..........................................................aaaUcugaacaaacuuuuuggc..............................   637     1    seq
    ..........................................................aaacGugaacaaacuuuuuggc..............................   1       1    seq
    ..........................................................aaaccugaacaaacuuuuuggc..............................   7       0    seq
    ............................................................aUcugaacaaacuuuuuggc..............................   16      1    seq
    ............................................................aUcugaacaaacuuuuuggcc.............................   73      1    seq
    .............................................................Ucugaacaaacuuuuuggcc.............................   30      1    seq
    .............................................................Ucugaacaaacuuuuuggcca............................   3       1    seq
```
